# Biomimetic Cuspal Rehabilitation Using Indirect Ceramic Onlay After Pulpal Preservation: A Case Report

**DOI:** 10.1155/crid/5817747

**Published:** 2026-10-09

**Authors:** Ihtisam Alam, Aravind R. Kudva, S. Vidhyadhara Shetty, Aboobacker Sidheeque

**Affiliations:** ^1^ Department of Conservative Dentistry and Endodontics Yenepoya (Deemed to Be University) Dental College Mangaluru Karnataka India

**Keywords:** immediate dentin sealing, indirect ceramic onlay, vital pulp therapy

## Abstract

**Background:**

Preservation of pulp vitality after pulpal exposure using vital pulp therapy (VPT) is now considered a predictable alternative to root canal treatment for normal or reversible pulpitis. With the growing demand for minimally invasive dentistry, indirect ceramic onlays offer good esthetic outcomes and durability with minimal tooth reduction.

**Case Report:**

A mandibular left first molar with deep caries was treated with direct pulp capping using mineral trioxide aggregate (MTA). Following initial setting of MTA, immediate dentin sealing (IDS) was performed, and tooth was prepared for an indirect ceramic onlay and was adhesively luted. At 1‐year follow‐up, the tooth presented normal response to pulp sensibility tests and absence of clinical or radiographic pathology.

**Conclusion:**

MTA‐based direct pulp capping combined with IDS and adhesively bonded ceramic onlay can provide a biologically and mechanically sound alternative to root canal treatment for selected permanent molars with deep caries having pulpal exposures.

## 1. Introduction

With the fundamental change toward minimally invasive biological treatment modalities, vital pulp therapy (VPT) has been receiving increasing attention in the management of deep caries. It involves the removal of damaged or infected parts of the pulp tissue, followed by the application of bioactive materials to promote repair, ultimately aimed at restoring the tooth′s function and esthetics [1]. Systematic reviews and randomized clinical trials indicate that calcium silicate cements such as MTA show higher success rates than calcium hydroxide when used as a direct pulp capping agent, especially after adequate hemostasis and coronal sealing [2, 3].

With the advancement in adhesive dentistry, immediate dentin sealing is a crucial step for the success of indirect restorations. It helps in tissue conservation, decreases bacterial contamination and marginal leakage, pulpal protection, and also maximizes the bond strength with the indirect restoration [4].

Collectively, these vitality‐preserving, tissue‐conserving, and adhesively bonded strategies constitute a biomimetic approach, defined as restorative management that seeks to reproduce the biological, mechanical, and esthetic behavior of natural tooth while conserving sound tooth structure and pulp vitality. So, this case report presents the combined use of MTA for direct pulp capping and IDS prior to indirect ceramic onlay placement in a mandibular first molar with deep caries showing favorable clinical and radiographic outcomes.

## 2. Case Presentation

A 22‐year‐old female patient reported to the Department of Conservative Dentistry and Endodontics, Yenepoya Dental College with the complaint of dislodged restoration in the lower left back tooth for the past 2 months. The patient had no history of lingering night pain or swelling. Medical history was noncontributory. Intraoral examination revealed an extensively carious mandibular left first molar (Tooth 36) with a deep occlusal‐proximal lesion extending toward the pulp chamber (Figure 1a). The tooth was nontender to percussion or palpation, and periodontal probing depths were within normal limits with no evidence of sinus tract or mobility. Thermal and electric pulp sensibility tests showed a normal response when compared with control teeth. A preoperative periapical radiograph revealed a deep carious lesion approximating the pulp in Tooth 36, with no evidence of periapical radiolucency or root resorption (Figure 1b). The diagnosis was reversible pulpitis with normal apical tissues and VPT was chosen as the primary treatment option instead of root canal treatment followed by an adhesively bonded indirect ceramic onlay as the definitive restoration.

**Figure 1:**
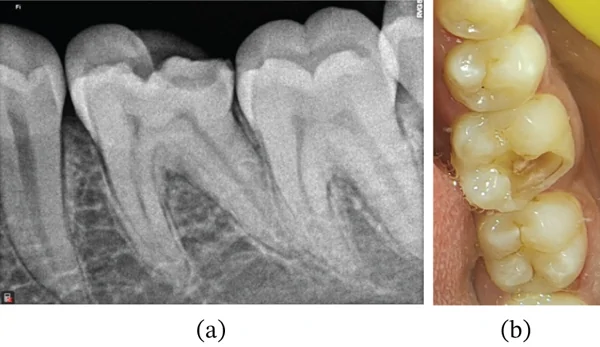
Preoperative status: (a) radiograph and (b) clinical photo of Tooth Number 36.

The tooth was anesthetized with an inferior alveolar nerve block using local anesthesia (Indoco Warren Lignox Lignocaine 2%, Indoco Remedies Ltd., India) and isolated with a rubber dam.

Caries removal was done using diamond rotary instruments under copious water spray, followed by hand excavation in the deepest areas to minimize mechanical trauma to the pulp. During caries removal, pulp exposure of approximately 2 mm occurred at the distal pulp horn (Figure 2a). Hemostasis was achieved within 5 min using 3% sodium hypochlorite.

**Figure 2:**
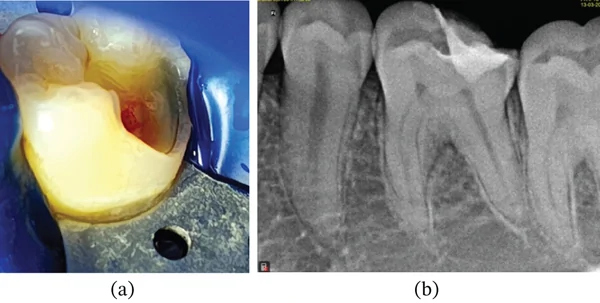
(a) Pulpal exposure and (b) immediate dentin sealing.

Then MTA (MTA Angelus, Angelus industrial de produtos odontalogicos, Brazil) was mixed according to the manufacturer′s instructions and was carefully placed over the exposure site with an approximate thickness of 2 mm. A moist cotton pellet was placed over the MTA for initial setting.

Since the exposure occurred at the completion of caries excavation, the exposure site was circumscribed by caries‐free dentin and was not in contact with residual infected dentin. Also, the tooth was preoperatively free of spontaneous or lingering pain and responded normally to thermal testing. So, direct capping was preferred in order to conserve the maximum volume of vital coronal pulp.

After 15 min, the dentin was immediately sealed using one‐step self‐etch (SE) adhesive system (Tetric N‐Bond Universal, Ivoclar Vivadent, Leichtenstein), which was applied to the cavity and light cured for 20 s, followed by placement of flowable composite resin (Figure 2b) (Tetric N‐Ceram, Ivoclar Vivadent, Leichtenstein). The cavity was then refined using diamond rotary instruments under copious water spray to receive the indirect restoration (Figure 3). An elastomeric impression was made and shade selection was performed. The tooth was provisionally restored using a resin‐based provisional material (3M ESPE Protemp 4 Temporisation Material, Germany) after isolating the cavity with Vaseline at the periphery and over axial walls, leaving a small central area for spot etching and bonding.

**Figure 3:**
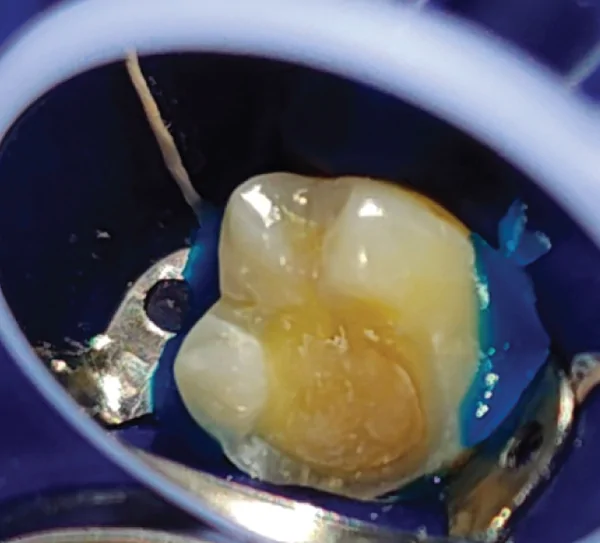
Prepared cavity.

A lithium disilicate ceramic onlay was designed and fabricated by the dental laboratory based on the impression and shade prescription (Figure 4). After receiving the fabricated onlay from the lab (Figure 5a), cementation appointment was scheduled where the provisional restoration was removed and the prepared tooth was cleaned with pumice. The intaglio surface of the onlay was etched with 9% hydrofluoric acid for 20 s (Figure 5b), rinsed, dried, and treated with a silane coupling agent and kept for 60 s. The enamel margins were selectively etched with phosphoric acid, and self‐adhesive resin cement (3M RelyXU200, Germany) was applied to the intaglio surface; the onlay was seated under controlled pressure and tack cured. Excess cement was removed, and light curing was performed from all accessible aspects according to manufacturer instructions. Occlusion was checked in centric and excursive movements, and minor adjustments were finished and polished (Eve DiaPro Twist, Germany). Then, postoperative clinical photograph was taken (Figure 6). The patient was instructed about postoperative care and was advised to report any symptoms such as spontaneous pain, swelling, or prolonged sensitivity.

**Figure 4:**
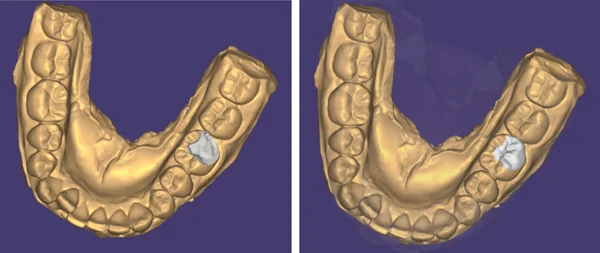
Fabrication of onlay using CAD/CAM.

**Figure 5:**
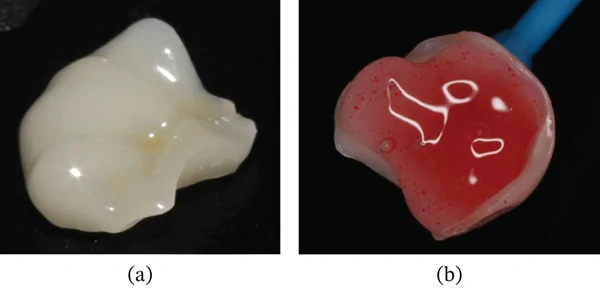
(a) Fabricated onlay and (b) conditioning of intaglio surface.

**Figure 6:**
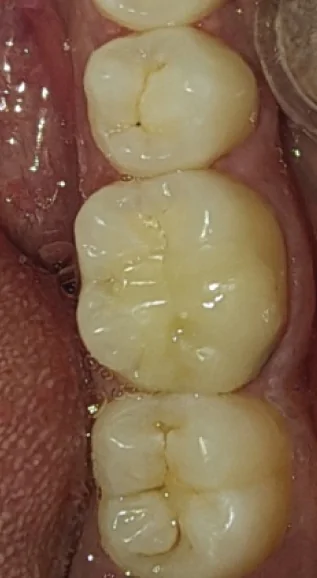
Postplacement clinical image.

At 1‐year follow‐up, clinical and radiographic evaluation showed a symptom‐free tooth, with no tenderness to percussion or palpation, with normal probing depths, and an intact restoration without any marginal discoloration, fracture or debonding (Figure 7). Thermal and electric pulp sensibility showed normal response, indicating maintained pulp vitality. There were no changes in the root canal space and lamina dura was seen intact radiographically.

**Figure 7:**
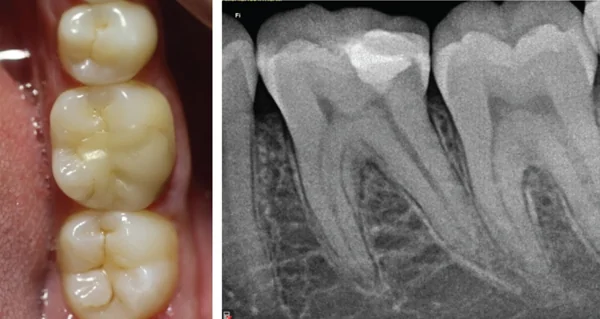
12‐month follow‐up clinical image and radiograph.

## 3. Discussion

This case illustrates that proper diagnosis and case selection, along with the selection of bioactive material and integrity of coronal restoration play a crucial role in successful VPT outcomes. As contemporary randomized trials and meta‐analysis consistently show superior success for MTA and other hydraulic calcium silicate cements compared with calcium hydroxide in permanent teeth as pulp capping and pulpotomy agents [2, 5, 6].

Resin coating technique and IDS are especially indicated in cases of indirect restoration on vital teeth, as pulp is indirectly exposed due to the presence of dentinal tubules, connecting it to the exposed surface. The rationale behind using the IDS technique for sealing freshly prepared dentin is to prevent the collapse of collagen fibers within the hybrid layer [4]. Filled DBAs or combinations with flowable RBCs are preferred to protect the IDS layer from conditioning procedures [7]. As evidence has shown, moderate and thick IDS layers act as a stress breaker under cyclic load stress when compared with thin or no IDS [8].

On the other hand, onlays offer improved esthetics, an adhesive bond to enamel and dentin that preserves the natural tooth structure, hence increasing the longevity of the tooth. Systematic review and meta‐analysis have shown that onlays fabricated using lithium disilicate ceramic have favorable fracture resistance and clinical outcomes in selected posterior teeth [9].

The choice between a direct composite restoration and an indirect ceramic onlay in such extensively compromised molars merits consideration. Contemporary evidence indicates that well‐executed extended direct posterior composite restorations can achieve comparable survival to that of indirect restorations, and a direct approach would be more economical. Moreover, it could have been completed in a single visit, and would have simplified any future endodontic access. However, in the present case, the extensive loss of coronal tooth structure with a compromised marginal ridge and reduced cuspal support favored a restoration providing cuspal coverage. An adhesively bonded lithium disilicate onlay was selected because it offered predictable cuspal protection, superior wear resistance and marginal adaptation across the wide proximal box, anatomically accurate proximal contacts and occlusal morphology developed extra‐orally, and avoidance of the polymerization shrinkage stress associated with placing a large volume direct composite and thereby reinforcing the weakened tooth in a biomimetic manner.

Although the present case demonstrates favorable short‐term outcome at 1 year, several limitations warrant consideration. The principal advantage of this combined approach lies in its biologically conservative philosophy: By preserving pulp vitality through MTA capping and protecting the exposed dentin through immediate sealing, the need for root canal treatment and the associated loss of tooth structure and fracture resistance may be avoided. However, late pulpal complications including delayed necrosis, internal resorption, or calcific metamorphosis cannot be excluded with only 1 year of follow‐up, as such sequelae may manifest several years after pulp exposure and capping. Similarly, restorative failures such as marginal microleakage, secondary caries beneath the onlay margins, or debonding at the resin‐dentin interface may only become evident with extended functional loading over time.

Although each individual component of this protocol, direct pulp capping with MTA, immediate dentin sealing, and lithium disilicate onlay restoration is well established in the literature, the present case contributes to existing evidence by demonstrating their integrated, sequential application within a single clinical episode in a posterior tooth with deep caries and pulp exposure, a scenario in which root canal treatment is still frequently the default approach. This case adds to the limited body of clinical documentation supporting a fully vital pulp preserving, minimally invasive pathway from exposure management through to definitive indirect restoration as a viable alternative in carefully selected cases, while underscoring the need for larger case series and longer term controlled studies to establish the durability of this combined approach beyond the short‐term follow‐up reported here.

## 4. Conclusion

Within the limitations of a single case with 1‐year follow‐up, the combined use of MTA direct pulp capping, immediate dentin sealing, and an adhesively bonded lithium disilicate onlay preserved pulp vitality and restored function in a permanent molar with deep caries having pulpal exposure, while conserving sound tooth structure. This minimally invasive, pulp‐preserving approach may represent a viable alternative to root canal treatment and full coverage restoration in carefully selected cases; however, the favorable outcome reported here reflects only short‐term observation, and longer term follow‐up together with larger controlled studies is required to confirm the durability of both the pulpal and the restorative results.

## Author Contributions

Ihtisam Alam: investigation, data curation, and writing—original draft. Aravind R. Kudva: conceptualization, supervision, and writing—review and editing. S. Vidhyadhara Shetty: supervision. Aboobacker Sidheeque: writing—review and editing.

## Funding

No funding was received for this manuscript.

## Disclosure

All authors have read and approved the final version of the manuscript. As the corresponding author and manuscript guarantor, I had full access to all the data in this study and take complete responsibility for the integrity of the data and the accuracy of the data analysis.

## Ethics Statement

Ethical approval was not required because this manuscript reports a single anonymized clinical case. Written informed consent for treatment and publication was obtained from the patient.

## Consent

This reporting case follows the CARE guidelines, and written informed consent was obtained from the patient. No data on the paper reveals the patient′s identity.

## Conflicts of Interest

The authors declare no conflicts of interest.

## Data Availability

The data that support the findings of this study are available on request from the corresponding author. The data are not publicly available due to privacy or ethical restrictions.
